# Metals and Alloys*Erratum.—P. 358. Sixteen lines from bottom, for “fully,” read “feebly.”

**Published:** 1862-12

**Authors:** B. Wood

**Affiliations:** New York


### DENTAL REGISTER OF THE WEST.

VOL. XVI.] DECEMBER, 1862. [No. 12.

### Original Essays and Communications.

### METALS AND ALLOYS.*

BY DR. B. WOOD.

(Continued from page 361, Vol. XVI.)

Before particularizing alloys it may be well to notice the metals whose combinations we propose to speak of.

TIN.—This metal is so well known that it would hardly appear necessary to describe it here. It is one of the most brilliant of metals,—color white with a slight yellowish tint; when oxidized the surface turns yellowish. It is exceedingly flexible. Subjected to flexion or torsion, it gradually attenuates, until the point of separation is reduced to a mere strand. It is one of the softest of the metals and stands on the scale but one degree harder than lead. When bent it emits a creaking sound, known as the "cry of tin," a peculiarity not possessed by other metals except cadmium and by this in a less perceptible degree. It is the most fusible metal in common use.

It tarnishes on the surface when left exposed to the air, but less than lead or bismuth or even cadmium.

It dissolves or rather oxidizes rapidly, with effervescence in

*Erratum.—P. 358. Sixteen lines from bottom, for "fully," read "feebly."

nitric acid, hot or cold, and is precipitated in the form of oxide ("stannic acid"). *It also dissolves freely in hot muriatic acid, but without commotion; the acid losing its yellowish hue and becoming limpid. Hot sulphuric acid acts upon it energetically with effervescence. In cold sulphuric acid it tarnishes upon the surface immediately, but the solvent action of the acid is quite feeble. The action of cold muriatic acid is also feeble; caustic potash in solution blackens and dissolves it. It is slightly affected by most of the vegetable acids. The action of acetic acid upon it is facilitated by heat.

"Tin is dissolved in a mixture of one part bitartrate of potash, two parts of alum, two of common salt, and a certain quantity of water" (Graham). This solution is used for tinning pins; and may frequently prove useful in the dental laboratory.

It is precipitated in a spongy state from its solution in muriatic acid by metallic zinc, the muriatic solution being converted, by substitution of zinc for tin, into muriate or chloride of zinc,—a liquid much used of late by some dentists for mixing up with oxide of zinc into a paste for filling teeth, forming the cement known as osteoplasty, bone filling, etc.

I will add a few excerpts from authors, which may serve to refresh the memory.

Tin combines with sulphur and with phosphorus, forming solids of a metallic appearance. Phosphorus, thrown on melted tin, will take up 15 to 20 per cent. and form a silver white phosphoret, soft enough to cut with the knife, somewhat ductile, but little malleable.

Tin, says Brande has only one ore, the native oxide, or tin stone; it has two oxides, the gray and the white; these are soluble in fixed alkalies.

*In dissolving tin in nitric acid the acid should be diluted as it is not affected by non-hydrate nitric acid, but on adding a little water the action is vehement.

Stream tin stone is found in Europe only in Cornwall, Eng., and is absolutely free from arsenic. It affords 65 to 75 per cent. of the very best and purest grain tin (Artist's Manual.)

The tin of Commerce is contaminated particularly by arsenic and tungsted. The Banca tin is the purest. Peruvian tin is quite impure, being used much for dental purposes, and as it is difficult to discriminate between the different kinds in market, it may be of some interest to know how to refine the metal.

To obtain it pure, reduce the tin to filings or granulate it by melting and pouring it in a small stream into water, and oxidize in an excess of nitric acid. Wash the oxide thus produced with hydrochloric acid, and then with water. Finally, reduce the oxide at a low white heat in a crucible lined with charcoal.

To purify Peruvian tin, Phillips, in his *Metallurgy*, gives the following directions: Fuse the tin in an iron pot and granulate by running off in cold water. Subject this to hot hydrochloric acid, the tin kept in excess. This results in protochloride, the tungsted being left in black powder. Pour the clear solution into a vessel containing a small quantity of the same impure tin, by which the remaining traces of arsenic and antimony are precipitated in black powder. Lead is removed by addition of sulphate of zinc or sulphuric acid. From this clear solution the purified metal is obtained by the introduction of plates of metallic zinc, whilst a solution of pure chloride of zinc is produced. The spongy metal thus thrown down is washed in dilute hydrochloric acid, and then in water, and fused in an iron pot;—pure as grain tin.

As a component of alloys, tin is one of the most useful of the metals. It lowers the melting point of copper and makes it susceptible of taking sharp impressions in casting, and with it forms the hardest and strongest alloys. It renders lead and bismuth and their compounds more fusible, and the alloys thus formed are much harder than either of their constituents. A small proportion of bismuth renders tin brittle. The effect of tin on alloys of bismuth and lead, in whitening them and preventing their tarnishing in the atmosphere, is remarkable. Lead, as is well known, blackens almost immediately in the air. Bismuth also tarnishes quickly, though less than lead. The compound, of these two metals are remarkable for undergoing oxidation in the atmosphere, so that after awhile quite a thick crust of oxide is formed on the surface. Tin, even in very small proportion, prevents this, while imparting a clean, white color to the alloy. Cadmium has a similar effect; these two metals, conjointly united with bismuth and lead, give the compound a pure silver white hue, and lower the melting point in a most extraordinary manner.

Tin of late years has taken the place of lead as a cheap material for filling teeth, on account of its being harder and less susceptible to tarnish in the mouth. Alloyed with about equal parts of silver, it is also much used for forming amalgams for tooth filling. Its uses in the laboratory are too well known to need mention,—or until we come to speak of alloys.—Hardness 2. Flex. 5. Mall. 5. Fus. 4.—45. (Table p. 215.)

In closing this paper, I must ask indulgence. Omissions there are, and errors there may be. In moving, my notes were thrown into confusion, and I have been compelled to rely mostly upon memory, with thoughts for the present preoccupied. Add to this the necessity of writing with haste, and doubtless it will give ample margin for criticism. Should I discover errors, I will correct them in the form of errata. For the rest they must take their chances.

New York, October, 1862.

(*To be Continued.*)

